# Humans Vary, So Cardiac Models Should Account for That Too!

**DOI:** 10.3389/fphys.2017.00700

**Published:** 2017-09-21

**Authors:** Barbara Wiśniowska, Zofia Tylutki, Sebastian Polak

**Affiliations:** ^1^Pharmacoepidemiology and Pharmacoeconomics Unit, Faculty of Pharmacy, Jagiellonian University Medical College Krakow, Poland; ^2^Simcyp Certara, Sheffield, United Kingdom

**Keywords:** variability, cardiac models, IVIVE, drug cardiac safety, modeling and simulation

## Abstract

The utilization of mathematical modeling and simulation in drug development encompasses multiple mathematical techniques and the location of a drug candidate in the development pipeline. Historically speaking they have been used to analyze experimental data (i.e., Hill equation) and clarify the involved physical and chemical processes (i.e., Fick laws and drug molecule diffusion). In recent years the advanced utilization of mathematical modeling has been an important part of the regulatory review process. Physiologically based pharmacokinetic (PBPK) models identify the need to conduct specific clinical studies, suggest specific study designs and propose appropriate labeling language. Their application allows the evaluation of the influence of intrinsic (e.g., age, gender, genetics, disease) and extrinsic [e.g., dosing schedule, drug-drug interactions (DDIs)] factors, alone or in combinations, on drug exposure and therefore provides accurate population assessment. A similar pathway has been taken for the assessment of drug safety with cardiac safety being one the most advanced examples. Mechanistic mathematical model-informed safety evaluation, with a focus on drug potential for causing arrhythmias, is now discussed as an element of the Comprehensive *in vitro* Proarrhythmia Assay. One of the pillars of this paradigm is the use of an *in silico* model of the adult human ventricular cardiomyocyte to integrate *in vitro* measured data. Existing examples (*in vitro—in vivo* extrapolation with the use of PBPK models) suggest that deterministic, epidemiological and clinical data based variability models can be merged with the mechanistic models describing human physiology. There are other methods available, based on the stochastic approach and on population of models generated by randomly assigning specific parameter values (ionic current conductance and kinetic) and further pruning. Both approaches are briefly characterized in this manuscript, in parallel with the drug-specific variability.

## Introduction

### Mechanistic modeling and simulation approach in the process of drug development in light of the recent changes to FDA/EMA/PMDA regulations

The mathematical modeling and simulation (M&S) approach has held its place in the drug development process since the very beginning. Having moved from academic curiosity to industrial practice the approach is used to both analyze the data and understand the physical mechanisms involved. Model-informed drug development (MIDD) is here to stay and it also has been recently indicated as one of the major areas of scientific priority by the regulators (Huang et al., 2013; Zineh et al., 2017). Initially, the outcome of pharmacometric analyses has impacted the decision making process of drug development (Miller et al., 2005; Lee et al., 2011). In recent years regulatory support for mechanistic physiological modeling has helped to bridge the gap between preclinical and clinical observations with respect to understanding biological systems (Rowland et al., 2015; Friedrich, 2016).

Special interest has been given to physiologically based pharmacokinetic (PBPK) modeling defined by WHO as “quantitative descriptions of the absorption, distribution, metabolism, and excretion (ADME) of chemicals in biota based on interrelationships among key physiological, biochemical and physicochemical determinants of these processes”^1^. A physiologically based pharmacokinetic approach is based on a combination of the physiology, environment, and drug specific information. These parameters are further utilized in the mechanistic models describing the pharmacokinetics (PK) and/or pharmacodynamics (PD) of a drug(s) of interest (Rostami-Hodjegan, 2012). This is not a new concept and it is suggested that the roots of PBPK models originate from the work of Teorell, published in 1937 (Teorell, 1937). Recent scientific advances and the development of models utilizing PBPK scaffolding are invaluable in the situation where clinical trials are extremely challenging or impossible. This includes specialized populations and situations of special interest such as pregnant woman and pediatric applications (Lu et al., 2012; Abduljalil et al., 2014). PBPK models are utilized for various applications throughout a drug's life cycle. Results of their simulations can be used to support the planning of specialized clinical studies, support dosing recommendations and the labeling of products (Zhao et al., 2011; Jones et al., 2015; Shepard et al., 2015; Wagner et al., 2015). The simulation results are used in lieu of conducting clinical studies or provide information that otherwise would have been missing in some specific situations (Jamei, 2016).

The use of PBPK modeling was included in the guidance documents for industry provided by the U.S. Food and Drug Administration (FDA), the European Medicines Agency (EMA) and the Ministry of Health Labor and Welfare (MHLW) of Japan. The latter, namely the draft of the drug interaction guideline for drug development and labeling recommendations published by the Japanese Ministry of Health, Labor and Welfare in 2014, suggested the PBPK application in the assessment of drug-drug interaction (DDI; Saito et al., 2014). Recent guidelines published by the FDA and EMA list several points in the drug development process where PBPK modeling may be applicable in order to support decisions in the premarketing, as well as at postmarketing, stage^2^^,^^3^. PBPK analyses are currently widely accepted and used not only as a research tool but also to support drug registration applications including investigational new drugs (INDs), new drug applications (NDAs), biologics license applications (BLAs), or abbreviated new drug applications (ANDAs; Sato et al., 2017). As of December 2016 there were 36 approved drugs, which provided PBPK M&S results, in the U.S. new drug application labeling procedure. In addition to PBPK, the Advisory Committee mentioned mechanistic safety modeling, particularly, risk prediction/assessment as a promising MIDD area^4^.

It is highly likely that the next application for PBPK models is in the area of precision dosing and personalized medicine (Hartmanshenn et al., 2016). This is achievable due to the specific structure of the physiologically-based models, where system description is clearly separated from the drug and external parameters (i.e., dosing schema). The biological parameters are described by large collections of anatomical and physiological data derived from literature or existing databases. For the assessment of inter- and to some degree intra-individual variability, virtual individuals, and virtual populations are randomly created (Jamei et al., 2009a).

### *In Vitro—in Vivo* extrapolation (IVIVE) as an approach

*In vitro—in vivo* extrapolation (IVIVE) as a phrase covers all techniques utilized for the prediction of human pharmacokinetics and pharmacodynamics based on the ADME information ADME in addition to drug activity and toxicity. It is crucial to mention that, in principle, the data comes from the *in vitro* studies, where various models and techniques are utilized. The main challenge is to translate the data, often heterogeneous, from the level of a “Petri dish” to the complex system of the human body. Therefore, what is needed are *in vitro* methods mimicking basic phenomena occurring in a human body at the cellular or subcellular level, and models describing the human body as a biological system. The latter describing models of human organs, tissues, or the whole body include the incorporation of the above mentioned PBPK systems. There are other approaches currently implemented, which combine and merge traditional compartmental models and various systems biology models, to describe biochemical and physiological phenomena (Sorger et al., 2011). It's worth adding that the addition of available human *in vivo* data enriches the model and can improve the degree of predictability (Tsamandouras et al., 2015). Practical utilization of the IVIVE concept, with the use of PBPK models and population data, covers various populations from healthy individuals up to the special populations (e.g., diseased—renal insufficiency, cirrhosis etc.) and pediatric populations (Sager et al., 2015).

Interestingly, conceptually similar approaches were independently introduced in the pharmacokinetics and pharmacodynamics area (Visser et al., 2014). The biophysically detailed models describing cell electrophysiology can be used for the *in vitro—in vivo* extrapolation, as proposed in the CiPA (Comprehensive *in vitro* Proarrhythmia Assay) initiative, as described below (O'Hara et al., 2011; Gintant et al., 2016). There is although one difference between the IVIVE/PBPK approach and the proposed IVIVE/safety approach. The latter, namely safety *in vitro—in vivo* extrapolation, has so far been done with the use of models describing cell electrophysiology without accounting for variability.

### CiPA initiative—what does it change, where we are, and what is potentially lacking

Regardless of the opinion on the current changes—whether it is a logical consequence of the evolutionary changes or Copernican Revolution—we are witnessing a significant paradigm shift in the area of drug cardiac safety assessment. The recently proposed CiPA schema for the preclinical evaluation of proarrhythmic liabilities proposes to assess proarrhythmic risk based on *in silico* reconstructions of human ventricular electrical activity. A biophysically detailed model aims to analyze repolarization abnormalities such as EADs. The input information includes *in vitro* measured data on the drug concentration-dependent inhibition of multiple human cardiac currents (Colatsky et al., 2016; Gintant et al., 2016). The O'Hara-Rudy (ORd) model was chosen as the starting point for developing an *in silico* model with the ultimate aim of providing a system suitable for regulatory decision making (O'Hara et al., 2011). The model is being further developed by the addition of a more mechanistic description of drug-channel binding kinetics (Li et al., 2017). The model proposes to generate output allowing for the separation of the reference drugs into three distinct risk categories—low, intermediate and high risk. The validation is planned to be performed with the use of 28 (12) drugs as detailed in the report presented at the FDA Briefing Document Pharmaceutical Science and Clinical Pharmacology Advisory Committee Meeting March 15, 2017 Topic: Strategies, approaches, and challenges in model-informed drug development (MIDD). According to the FDA's suggested strategy, estimation of the inter-individual variability in a drug's pharmacokinetics and pharmacodynamics is a key issue in recent drug development. PBPK modeling addresses this issue and so should mechanistic safety modeling. In PBPK, the variability in PK prediction is assured by specifying the population-dependent distribution of parameters' values and the covariation between these parameters (Jones et al., 2015). Regarding cardiac risk assessment, the variability is already observed at the stage of *in vitro* measurements, e.g., in drug effects on the ventricular ion currents or in the effects on human stem cell-derived cardiomyocytes (iPSC-CM) which differ in channel gene expression profiles and patterns of arrhythmic events after testing with the same model drug (Elkins et al., 2013; Blinova et al., 2017). The observational uncertainty together with other sources of uncertainty influence computational model inputs, and consequently, the confidence of the output, regardless of the electrophysiological model used (Johnstone et al., 2016; Mirams et al., 2016). The frequently used cardiac models do not account for physiological or experimental variation in their default parametrization (Davies et al., 2016). However, clinical observations leave no doubts that variability is important, and drug-independent factors may play a crucial role in triggering a drug cardiac effect. The analyses of case reports of QT interval prolongation and ventricular arrhythmia, associated with cisapride, revealed that often in the case of these adverse events the patients had more than one contraindication that predisposed them to arrhythmia. The drug was therefore withdrawn from the U.S. market in 2000 (Wysowski et al., 2001). The coincidence of multiple risk factors, both physiological and pathophysiological was also the case for TdP induction after the administration of certain drugs, e.g., erythromycin (Hancox et al., 2014), quetiapine (Hasnain et al., 2014), methadone (Vieweg et al., 2013b), and risperidone (Vieweg et al., 2013a). These are all exemplary drugs which are known to pose the risk of TdP according to the Credible Meds classification and are at the same time safely used if taken properly^5^. The observed variability in the PD response may come from the drug itself, since for some compounds the QTc interval prolongation is said to be dose or concentration-related (Krantz et al., 2003; Fanoe et al., 2009). However, even then the variation in individual QTc length cannot be explained solely by PK and factors that affect the ADME processes. There are examples illustrating the lack of correlation between electrophysiological changes and drug plasma concentrations (Wiśniowska et al., 2016). Even in healthy subjects the observed QTc changes, following drug administration, may vary by about 80 ms (Jerling and Abdallah, 2005; Hulhoven et al., 2008) as age, sex, and race are said to affect cardiac electrophysiology (Macfarlane et al., 1994) not to mention observed circadian intra-subject variations (Molnar et al., 1996).

There are also special populations that should be considered when assessing drug triggered cardiac effects. First of all, the cardiac action potential is affected in patients with cardiac channelopathies associated with genetic mutations. One of the main congenital phenotypes is long QT syndrome (LQTS) which is prevalent in 1:3,000–1:5,000 in the general population (Goldenberg and Moss, 2008) manifesting with a QTc length of above 460 ms (Abriel and Zaklyazminskaya, 2013). The most frequent mutations that are responsible for congenital LQTS were found in genes that code for proteins in the potassium hERG channel (KCNQ1 and KCNH2) and the Nav1.5 sodium channel (SCN5A; Bohnen et al., 2016). The LQTS patients are said to be particularly vulnerable to drug-related arrhythmias (Goldenberg et al., 2008). Also, comorbidities were found to contribute to QTc-prolongation. The analysis conducted by Vandael et al. (2017) revealed strong evidence for ischemic cardiomyopathy, hypertension, arrhythmia, and thyroid disturbances to be risk factors contributing to QTc interval prolongation.

### Variability in cardiac models—stochastic vs. deterministic approach

Mathematical models of cardiac cell electrophysiology have proved their value and now hold an established position in research and drug development (Amanfu and Saucerman, 2011; Davies et al., 2016). It all began with the work of Hodgkin and Huxley where they modeled the cell membrane as a capacitor with batteries and resistors and described its electrophysiological behavior (Hodgkin and Huxley, 1952). The following ordinary differential equation was established:

$$\begin{array}{l} \frac{dV}{dt}=\frac{I_{ion}-I_{stim}}{C_{m}}, \end{array}$$

where V is voltage, t is time, I_ion_ is the sum of all transmembrane ionic currents, I_stim_ is the externally applied stimulus current, and C_m_ is cell capacitance of the membrane per unit surface area (Ten Tusscher and Panfilov, 2006). Beginning from the relatively simple Noble's model (Noble, 1962), cardiac electrophysiology models have evolved tremendously, and now include a detailed description on cardiac ion channels, pumps and transporters, as well as intracellular calcium handling (Noble, 2007; Fink et al., 2011). The models proved successful for studying cardiac physiology and understanding pathological changes (e.g., arrhythmias) associated with diseases over the last decades and currently they are used in the safety assessment of drugs (Mirams et al., 2012; Roberts et al., 2012; Davies et al., 2016; Gintant et al., 2016). To be a vital element of the safety-related decisions made through the various stages of drug development and in the clinic, the models have to ascertain credibility, i.e., properly reproduce the electrophysiology of a population and individual patients. The question arises asking if it is feasible to use a single traditional cardiac model with input parameters being the mean values, averaged across many subjects, and generating an output as a single value, thus presenting behavior a of “representative” cell. Until recently there wasn't much interest in the variability in the field of cardiac electrophysiology modeling. Several approaches have now been proposed, and implemented, to introduce variability into cardiac models and account for inter- and intra-patients differences. All of the approaches stem from the belief that the “average patient” does not exist and a traditional model cannot accurately explain the observed differences between patients.

### Stochastic approach

Physiological variability can be investigated and modeled by constructing populations of experimentally calibrated models. This approach has been introduced a while ago and is further developed by researchers from various organizations including, but not limited to, the Department of Pharmacology and Systems Therapeutics, Mount Sinai School of Medicine, New York (Sobie, 2009; Sarkar et al., 2012), the Department of Computer Sciences at Oxford University (Britton et al., 2013; Sánchez et al., 2014; Muszkiewicz et al., 2016; Pueyo et al., 2016; Zhou et al., 2016), and other (Marder and Taylor, 2011). Variability in a model's behavior is accomplished by the multiplication of its parameter values by sampled scaling factors, which results in an ensemble of possible outputs instead of an average one. Once the baseline model (appropriate for the research question) is selected, the parameters of variation have to be chosen, and the ranges for parameter sampling need to be defined. Both depend on the study aim and the target population (e.g., healthy, diseased) to be investigated.

There are multiple approaches available for generating the population of models. For example, parameters and their ranges generate high-dimensional spaces from which their values are sampled using different sampling algorithms (e.g., the Latin Hypercube, Monte Carlo method) to generate an ensemble of variant models (Drovandi et al., 2016). The candidate models are simulated to mimic, for example, experimental settings regarding bath solution composition, voltage protocol, and pacing rate. The pool of models is pruned according to the boundaries for a range of permitted model outputs, defined by minimal and maximal values observed in the experiment. Such calibration yields the experimentally-calibrated populations of models presenting electrophysiological behavior that reproduce the results of experiments which can be further analyzed for the parameters underlying the observed response variability (Britton et al., 2013). The model could also be employed to assess a range of responses across the population under certain conditions, e.g., diseased, or reflecting drug application (Muszkiewicz et al., 2016).

The other approach is based on parameter sensitivity analysis techniques which can be applied to generate quantitative predictions based on considering behaviors within a population of models (Romero et al., 2009). In the single parameter scanning one can increase or decrease the parameter of interest, run simulation and save the simulation results. Such procedure can be performed for multiple parameters, with the use of various techniques and the outcome is expressed in a quantitative manner. Sobie and colleagues have also utilized more complex procedures and varied all parameters at once (Sarkar and Sobie, 2010, 2011). Assuming that the endpoint of interest is dichotomous in nature (i.e., EAD occurence) Morotti and Grandi proposed technique based on the multivariate logistic regression analysis, allowing for sensitivity analysis and investigating factors influencing the endpoint occurrence at the same time (Morotti and Grandi, 2016).

The methodology applied to experimentally-calibrated populations of models can be regarded as the refinement and extension of the sensitivity analysis method and populations of models constructed without a calibration step. In the latter two, values of single or multiple parameters are varied in a predefined range, while allowable values of model outputs are not constrained with experimental data thus may not be representative for certain subject groups. All of these models offer valuable insights into sources of variability and the pathological background of different heart conditions. Moreover, they allow the assessment of not only the average drug effect on cardiac electrophysiology, but also allow the screening of drug effects across a certain population. They, however, cannot be employed to evaluate the risk of an individual patient who is to be treated with a certain drug. This is because most variability sources considered by the model (e.g., ion channels conductance, gating kinetics, densities, resting membrane potential, membrane potential, upstroke velocity) are factors whose values cannot be determined for the particular patient. Another problem is the requirement to define the cut off threshold for non-physiological simulation results, since such a decision is always arbitrary.

### Deterministic approach

The other approach is based on the observation and description of the biological parameters characterizing the system of interest, in this case the human organism. Data describing demographic, genetic, anatomical, and physiological factors are collected and analyzed to describe the distribution of the parameter in a population and allow constructing its virtual counterpart instead of an average patient. Also some of those factors can be correlated with the parameters of a cardiomyocyte model, e.g., age and the cardiomyocyte volume and area (Polak et al., 2012). The main data sources remain the published scientific reports and clinical databases (ICRP, 2002; Valentin, 2003). This approach allows for differentiation between different populations; both healthy (e.g., considering ethnicity) and diseased (e.g., obese, renally impaired, diabetic), or of special interest (e.g., pregnant women, pediatric population; Jamei et al., 2009b). Equations describing the distribution of the system parameters for the PBPK model are derived from the distributions of data based on real populations and patients. Additionally, such an approach takes into account the dynamic changes observed with the parameter of choice (e.g., circadian changes of heart rate). An application includes the ontogeny description and age dependent variation of the chosen parameters (Salem et al., 2013). What is also of importance is the option to define the inter-correlation between various parameters. A virtual population can be generated from values and formulae describing demographic, anatomical, and physiological variables using a correlated Monte Carlo approach which protects from the non-physiological combinations of the model parameters (e.g., kidney size and liver size). This allows the prediction of variability before the clinical study phase, in contrast to a statistical approach (e.g., population PK analysis), which requires prior clinical data to characterize variability. Additionally it allows for the clear separation of information on the system (i.e., human body) from that of the drug (e.g., physicochemical characteristics), and the environment (e.g., dose, concomitant drugs).

It is obvious that the model prediction will depend on the quality of the gathered data, correctness of the analysis, and appropriateness of the conclusions drawn from the distribution of the parameters. Therefore, the criteria of inclusion and exclusion for the reports and papers (in addition to certain values) have to be defined prior to the commencement of the data collection process. The same applies to the statistical analysis, and all methods and tools.

There are multiple biological parameters influencing the ECG and its behavior. From a biological perspective, factors influencing cardiac electrophysiology can be classified into one of three key groups: (i) demography; (ii) anatomy, and/or physiology; and (iii) genetics. Examples of the most important parameters are listed below:

- Demography—age, gender
- Anatomy and/or physiology—plasma ions concentrations (K^+^, Ca^2+^), cardiomyocyte size (volume, area), cell electric capacitance, heart wall thickness, heterogeneity of cells across heart wall, heart rate, sex hormones
- Genetics—common polymorphisms and mutations at the level of ion channels/pumps/exchangers

For some of the above mentioned parameters statistical models describing their distribution in the population were developed based on the available data. This includes relationship between age, human left ventricle cardiomyocyte volume, and electric capacitance (Polak et al., 2012), left ventricular heart wall thickness (Fijorek et al., 2014a), or plasma ions concentration (Fijorek et al., 2014b). The latter, namely plasma ions concentration, together with the heart rate follow the circadian variability (Massin et al., 2000; Sennels et al., 2012). Such time of the day dependent variation influences the ECG and the parameters including QT (Karjalainen et al., 1994). Therefore, models describing the diurnal variability of such crucial parameters are also being developed and utilized (Fijorek et al., 2013a,b). They are also included in the virtual population generators, which build the virtual individuals being exposed to the drug in the *in silico* conditions (Mishra et al., 2014; Wiśniowska and Polak, 2016). The main limitation of this method is the limited amount of data available for the model building and information relating to the inter-correlation between parameters.

It is undisputable that sources of variability identified and already incorporated into *in silico* models cannot explain the whole observable inter- and intra-individual variability. The question is if it is already sufficient to provide reliable predictions.

### Drugs-specific variability

In the model-based drug safety assessment, apart from system-dependent variability, there is also another source of variability (or rather uncertainty) introduced into the model. The effects caused by exposure to a drug are modeled as concentration-dependent changes of ion currents known to be affected by the drug. The drug-channel interaction is quantified experimentally by constructing a Hill plot characterized by IC50 and the Hill coefficient value. There is no standardized protocol for drug-triggered ion channel block measurement, however multiple cell models and voltage protocols are accepted. This results in significant discrepancies between IC50 values reported for a given compound (amiodarone: 0.015–38.3 microM; cisapride: 0.0051–1.6 microM; dofetilide 0.003–25 microM; E4031: 0.001–15.8 microM; moxifloxacin: 0.93–398.1 microM; propafenone: 0.085–123 microM; to give just a few examples)^6^. Additional, uncertainty introduced into *in silico* models comes from uncertainty in the Hill coefficients of reported concentration-inhibition curves, especially when ion channel blocking potency for a compound is estimated by high-throughput screening methods (Elkins et al., 2013). Moreover, even the well-controlled experiments, carefully conducted in the same experimental settings, generate different results. This can be due to the measurement errors, intrinsic- and extrinsic-variability between samples. The subjective selection of IC50 values, which are taken as inputs into *in silico* models, may lead to misleading results. Drug-specific uncertainty is an undesirable type of variability, however infeasible to eliminate. The use of averaged values has been proposed to minimize drug-specific bias (Elkins et al., 2013). The question remains how, if possible, average results produced with different methods and experimental settings.

The situation is even more complex when the aim is to model a response to a drug in a realistic population where other drug-related factors like concomitant medicines, compliance, food and alcohol consumption have to be accounted for since all these factors can substantially influence the response.

## Conclusions

Drug pro-arrhythmic potency is a function of the intrinsic characteristics of the chemical structure and external parameters associated with the drug. The latter, namely external parameters, includes system-dependent and environment-dependent items. Therefore, to properly predict and assess the potential risk all significant elements should be considered. Translation of the *in vitro* data to an *in vivo* situation, e.g., to optimize clinical trials, requires such an approach. There are at least two ways to describe the population specific variability, as described above, and these elements should be accounted for in the *in silico* based cardiac safety assessment. On the other hand, the choice of an assessment method depends on the actual stage of the drug development cycle. In the early stages, including discovery phase such complexity is not necessary, but as the compound advances along the development process, the assessment should be more detailed and comprehensive.

It is worth noting that the diseased population would probably require even more parameters, and the variability would be larger, as compared against healthy individuals. This is because of the interrelation of parameters directly (e.g., cell volume and capacitance), and indirectly (e.g., thyroid-related diseases and fluid-electrolyte balance disruption) influence the heart cells electrophysiology. Examples of the above-mentioned parameters and their analysis clude hypertrophic cardiomyopathy (HCM) of various character (Polak and Fijorek, 2012). It is worth noting that stochastic approach, based on the virtually simulated population of models can be used to identify ionic mechanisms driving electrophysiological abnormalities not only in HCM, but also atrial fibrillation, and other diseases, accounting for of the disease specific variability (Liberos et al., 2016; Passini et al., 2016). Modeling and simulation approach can be also utilized to analyze the influence of genetic modifications at the level of ionic channels on the cardiac myocytes electrophysiology (Glinka and Polak, 2013). This includes the recently published example of the population of models optimized to recapitulate clinical long QT phenotypes (Mann et al., 2016). The detailed discussion of this element, namely the disease related variability is out of the current manuscript scope. There is however clear gap in all current approaches, namely comprehensive, or to be precise—as comprehensive as possible—parametrization of the disease of choice, including all known parameters. This would allowed to simulate population of virtual individuals closely mimicking those met in the clinical settings. The models usage could be than extended from the drugs' safety assessment to the drugs therapy optimization.

## Author contributions

BW, ZT, and SP equally contributed to the conception of paper, drafting the manuscript and approved its final version.

### Conflict of interest statement

SP is a Simcyp (part of Certara) employee. The other authors declare that the research was conducted in the absence of any commercial or financial relationships that could be construed as a potential conflict of interest.

## Footnotes

^1^www.who.int/ipcs/methods/harmonization/areas/pbpk/en/

^2^www.ema.europa.eu/docs/en_GB/document_library/Scientific_guideline/2016/07/WC500211315.pdf

^3^www.fda.gov/downloads/Drugs/GuidanceComplianceRegulatoryInformation/Guidances/UCM531207.pdf

^4^www.fda.gov/downloads/AdvisoryCommittees/CommitteesMeetingMaterials/Drugs/AdvisoryCommitteeforPharmaceuticalScienceandClinicalPharmacology/UCM544838.pdf

^5^https://crediblemeds.org/

^6^www.tox-database.net
